# Idiosyncratic learning performance in flies

**DOI:** 10.1098/rsbl.2021.0424

**Published:** 2022-02-02

**Authors:** Matthew A.-Y. Smith, Kyle S. Honegger, Glenn Turner, Benjamin de Bivort

**Affiliations:** ^1^ Department of Organismic and Evolutionary Biology and Center for Brain Science, Harvard University, Cambridge, MA 02138, USA; ^2^ Ann and Robert H. Lurie Children's Hospital of Chicago, Chicago, IL 60611, USA; ^3^ Janelia Research Campus, Howard Hughes Medical Institute, Ashburn, VA 20147, USA

**Keywords:** generalized learning, individuality, personality, *Drosophila*, Pavlovian conditioning

## Abstract

Individuals vary in their innate behaviours, even when they have the same genome and have been reared in the same environment. The extent of individuality in plastic behaviours, like learning, is less well characterized. Also unknown is the extent to which intragenotypic differences in learning generalize: if an individual performs well in one assay, will it perform well in other assays? We investigated this using the fruit fly *Drosophila melanogaster*, an organism long-used to study the mechanistic basis of learning and memory. We found that isogenic flies, reared in identical laboratory conditions, and subject to classical conditioning that associated odorants with electric shock, exhibit clear individuality in their learning responses. Flies that performed well when an odour was paired with shock tended to perform well when the odour was paired with bitter taste or when other odours were paired with shock. Thus, individuality in learning performance appears to be prominent in isogenic animals reared identically, and individual differences in learning performance generalize across some aversive sensory modalities. Establishing these results in flies opens up the possibility of studying the genetic and neural circuit basis of individual differences in learning in a highly suitable model organism.

## 1. Introduction

Genetically identical *Drosophila melanogaster*, raised in identical environments, display individuality in numerous innate behaviours [1–8], including light preference [2], left–right turning [3], temperature preference [4], postural behaviours identified by unsupervised analyses [5] and object-tracking [6]. Work to date has focused exclusively on innate or spontaneous behaviours. But plastic behaviours, such as learning, also have the potential to exhibit individuality, as each animal may have an idiosyncratic propensity to respond to training stimuli [9]. Individual variation in learning within insect populations has been described as early as 1907, by Charles Turner [10,11] in ants and honeybees. To our knowledge, individual variation in learning among genetically identical flies has not been characterized.

Here, we present evidence that genetically identical flies exhibit individuality in their ability to learn odour associations. Drawing inspiration from a classical Pavlovian conditioning assay [12–14], animals are exposed to two stimuli simultaneously, a so-called ‘conditioned stimulus' (CS+), to which their behavioural response will change across the conditioning, and a so-called ‘unconditioned stimulus' (US), to which their response will remain invariant [15]. In addition, flies were exposed to a second odour, the ‘CS−', without a US. The learned response to this training is likely to be avoidance of the CS+, as our US is aversive. Our experimental instrument, inspired by [9], allowed (i) measurement of individual learning performance, (ii) the automated selection of different CS odorants and (3) the use of electric shock or optogenetic activation [16] of negative valence neural circuit elements as US [17]. With this instrument, we can test a fly's generalized learning performance through reversal learning trials (i.e. swapping the CS+ and CS− odours). This paradigm represents a more cognitively demanding form of learning compared to classical conditioning because it requires modification of the previous association [18–23]. We also examined the generality of learning differences by training the same flies across two aversive US modalities: shock and optogenetic stimulation of bitter taste receptor neurons. We found positive correlations in learning performance when varying either the CS odorant or US. Thus, individual learning performance in flies appears to generalize across some stimuli.

## 2. Material and methods

All flies were grown on cornmeal/dextrose food in incubators (25°C, 40% relative humidity, 12 : 12 h light : dark cycle). Behaviour experiments were conducted on females 7–8 days post eclosion. For optogenetic experiments, *Gr66a-LexA* and *LexAop-CsChrimson* flies were crossed to produce experimental F1 s. Gr66a-LexAp65 was constructed using Sequence and Ligation Independent Cloning [24]. The Gr66a promoter fragment was the same 1798 bp segment used previously [25] and extended from the translation start site of the Gr66a open reading frame to the next upstream gene. This was joined to the start codon of the LexA::p65 transcriptional activator from pBPLexA::p65Uw [26] in a vector backbone derived from pUASTattB [27] by removing the UAS sites. The construct was integrated into the attP18 site. In experimental groups receiving the optogenetic US, 10 μl of 100 mM all-trans-retinal was applied to the surface of fly food, and flies were housed on this food for at least 48 h. Flies were aspirated directly into the behavioural arenas without anaesthetization.

**Figure 1.**
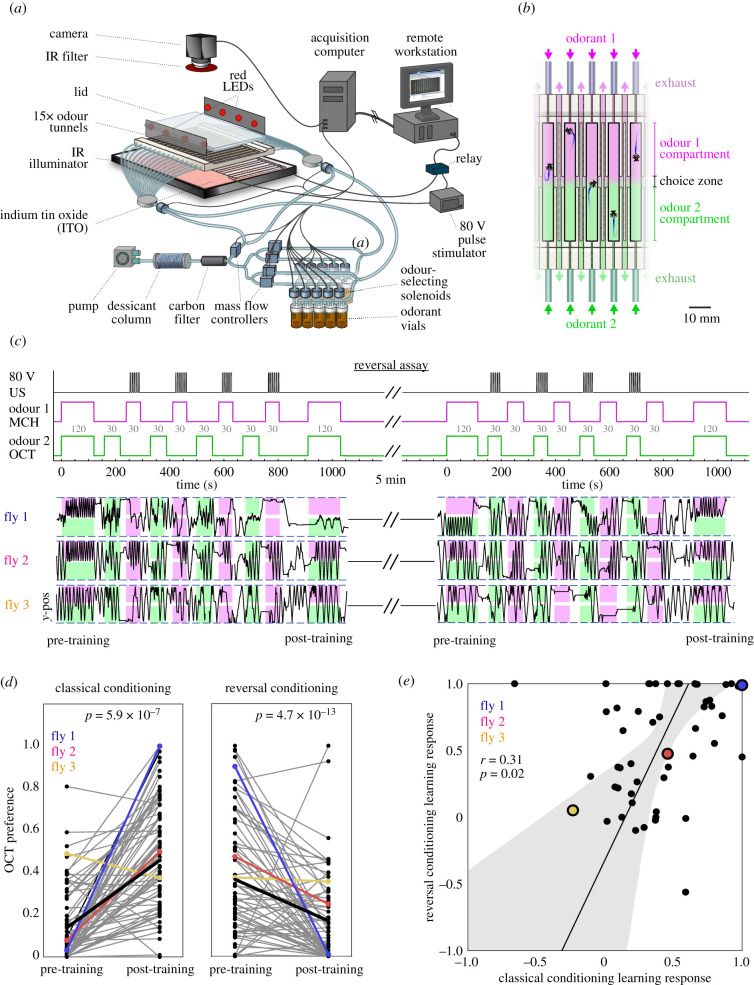
Individuality in associative learning. (*a*) Schematic of the reversal assay. (*b*) Zoom-in view of the linear behavioural arenas, with odorant flowing into each half. (*c*) Diagram of training protocol (top). Grey numbers indicate the length in seconds of each stimulus phase. Note that the timing of US delivery differs in the classical and reversal phases. Position in the arena versus time kymographs of three specific flies undergoing conditioning. Magenta and green shading indicate the portions of each arena that are filled with OCT and MCH, respectively. (*d*) Octanol preference of flies before and after training with MCH as the CS+ (left) and with OCT as the CS+ (right). Points are individual flies. Coloured examples correspond to the individual flies highlighted in (*c*). *p*-values reflect paired *t*-tests. Thick black line represents the mean. (*e*) Scatterplot of individuals’ learning responses for reversal versus classical conditioning trials (*r* = 0.31; *p* = 0.02; *n* = 53). Points are individual flies. Line is the best linear fit and shaded region is the 95% CI of the best-fit line.

**Figure 2.**
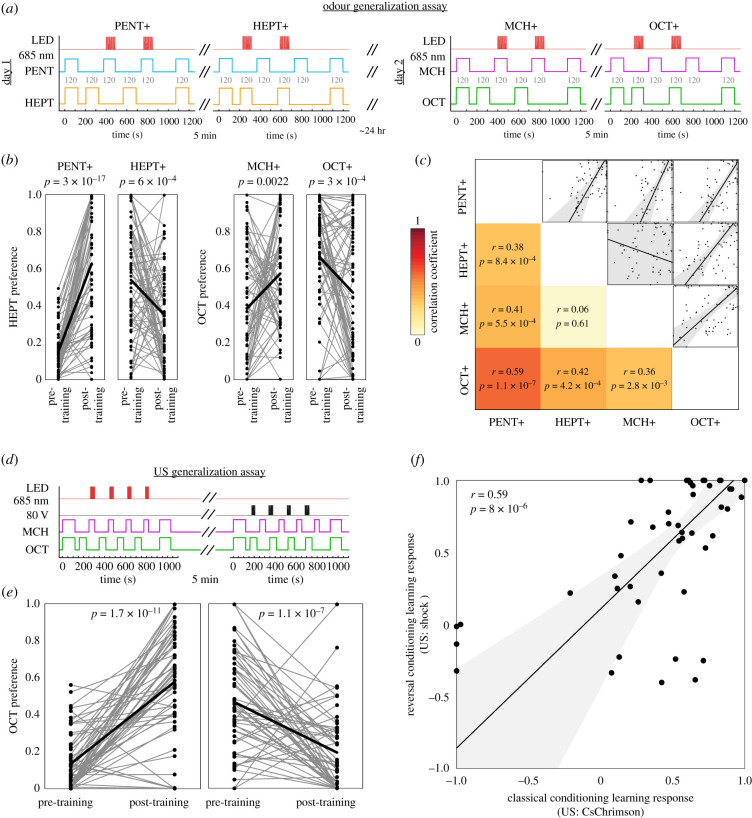
Individual learning across odours and US modalities. (*a*) Schematic of the odour generalization assay. Grey numbers indicate the length in seconds of each stimulus phase. (*b*) Odorant preference of flies before and after training for all the learning trials of (*a*). Odorant labels indicate the CS+ in each trial. Points are individual flies. Thick black line represents the mean. *p*-Values reflect paired *t*-tests. (*c*) Correlation matrix for individual fly learning responses for all pairs of learning trials in (*a*) and (*b*). *x*- and *y*-Axes of scatter subplots correspond respectively to the learning responses of the CS+ condition indicated by the column and row of the matrix. Points are individual flies. Line is the best linear fit, and shaded region is the 95% CI of the best-fit line. (*d*) Schematic of the US generalization assay. Stimulus phases have the same durations as in figure 1*c*. (*e*) Octanol preference of flies before and after training with shock as the US (left) or optogenetic activation of bitter taste neurons (right). Points are individual flies. Thick black line represents the mean. *p*-values reflect paired *t*-tests. (*f*) Scatterplot of learning responses to the shock US trial versus the bitter taste US trial (*r* = 0.45; *p* = 0.01; *n* = 47). Points are individual flies. Line is the best linear fit, and shaded region is the 95% CI of the best-fit line, suggests learning responses to HEPT may not be idiosyncratic.

The assay instrument consisted of 15 linear tunnels with inlets at either end and a vent at the centre (figure 1*a,b*). In each trial, a single fly was placed into each tunnel and allowed to walk freely. Laminar airflow carrying odour stimuli enters the tunnels from either end and meets at the centre, forming a sharp boundary. From there, the odorized air is vented to the room (figure 1*b*). Odorants were generated by flowing clean air over liquid odorants in a series of vials, under the control of solenoids and mass-flow controllers, as described in [8]. Within the arena, flies were presented one pair of odours (e.g. methylcyclohexanol [MCH] versus octanol [OCT] or 1-pentanol versus 2-heptanone [HEPT]). Shock US was delivered via laser-cut grids of indium tin oxide installed on each tunnel floor. Eighty volt direct current pulses from a Grass SD9 Pulse Stimulator (20 Hz for 5 s) were delivered at 10-s intervals. For optogenetic experiments, 626 nm red LEDs were used to activate CsChrimson and pulsed at 20 Hz for 3 s with a 5 s interstimulus interval. See assay timelines in figures 1 and 2. Behaviour was recorded using a CMOS camera (Point Grey Firefly MV) with a longpass filter (Kodak Wratten Filter 87C) at 60 Hz. Tracking was performed using custom MATLAB scripts that used two-dimensional cross-correlation for tunnel and initial fly identification, and background subtraction to locate fly centroids.

With this instrument, we implemented three associative conditioning assays. Flies were subject to exactly one of these assays, all of which were conducted in a temperature-controlled environmental chamber in darkness at 25°C and 40% relative humidity. The start of each assay consisted of a 2 min pre-training period in which the CS+ and CS− odorants were present in the tunnels, allowing us to measure flies’ untrained odour preference, which was variable as expected [8]. In the ‘reversal assay’, flies were first subject to classical pairing of odour with shock and then a reversal pairing in which the CS+ and CS− odorants were swapped. Learned responses were assessed after each pairing in a choice between the CS+ and CS− odours without shock (i.e. a single extinction trial). The ‘odour generalization assay' took the form of two reversal assays (using four odorants in total) conducted on successive days, between which the individual identity of flies in this assay was maintained by housing flies in modified 96-well plates (flyPlates, FlySorter, LLC; [28]). The ‘US generalization assay' took the form of the reversal assay but replaced electric shock with optogenetic bitter US in the initial classical pairing. See schematic of assay phases in figures 1 and 2.

Individual learning responses were measured by the normalized magnitude of change in occupancy towards the CS− from pre-training to post-training. This metric has a value of 0 if flies exhibit no learning, 1 if they spend all their time post-training in the CS− compartment and −1 if they spend all their time post-training in the CS+ compartment. Normalizing by the pre-training preference response accounts for individual variation in baseline preference [8].

$$\mathrm{learning}\;\mathrm{response}\;=\{\begin{array}{c} \frac{\mathrm{post}-\mathrm{pre}}{1-\mathrm{pre}}\;\mathrm{if},\;\;\;\mathrm{post}-\mathrm{pre}\;>\;0\\ \frac{\mathrm{post}-\mathrm{pre}}{\mathrm{pre}}\;\mathrm{if},\;\;\;\mathrm{post}-\mathrm{pre}\;\leq \;0 \end{array}$$

The correlation of learning responses across trials was calculated as the Pearson correlation coefficient, and all *p*-values are nominal. Data analysis was performed using custom MATLAB scripts. Raw data and analysis scripts [29] are available at http://lab.debivort.org/individuality-in-learning and https://zenodo.org/record/4458572.

## 3. Results

As expected, both training sessions in the reversal assay resulted in significant changes in mean OCT preference across flies (figure 1*d*). This mean change was not observed in control experiments (pairing the US with both CS+ and CS−, backward conditioning or presenting the CS alone; electronic supplementary material, figure S1). However, we also observed individual flies that appeared to not learn on a given trial, with similar preference for OCT pre- and post-training or increased OCT preference even when OCT was the CS+. These observations could reflect statistical noise, rather than individual variation in learning response. To test this, we examined the correlation between the learning response during the classical and reversal phases of the reversal assay. This correlation was positive and significant across individual flies (*r* = 0.31, *p* = 0.02; figure 1*e*), suggesting that individual animals have idiosyncratic learning responses that generalize across the identity of the CS+ odorant. (A few individuals exhibited the same odour preference after both classical and reversal phases, appearing to respond to one association but not the other, a result that could also be explained by these flies having a strong naive preferences that do not change across the assay.) Consistent individual differences in learning response were not correlated with a fly's activity (distance travelled) during the assay or initial odour preference (electronic supplementary material, figure S2), and we found no evidence that variation in learning could be explained by variation in prediction errors (electronic supplementary material, figure S1D).

The observation that individual performance following classical and reversal conditioning is correlated suggests that learning ability may generalize across sensory channels in flies. To explore this possibility, we implemented the odour generalization assay in which flies were subject to classical and reversal conditioning with 1-pentanol and 2-heptanone as CS odours, stored for 24 h and subject to classical and reversal conditioning with MCH and OCT (figure 2*a*). In addition, we substituted optogenetic stimulation of bitter taste neurons as the US instead of electric shock (electronic supplementary material, figure S3). This was done by expressing CsChrimson [16] in bitter taste neurons using a *Gr66a-LexA* driver and exposing flies to 626 nm LED illumination in place of the electric shocks. Replacing shock with bitter taste also let us assess whether individuality and correlation in learned responses to classical and reversal conditioning is US specific. In addition, by looking at learning performance after 24 h, we could assess whether individual variation in learning performance is stable over time. As we saw with shock–odour conditioning, flies subject to optogenetic bitter–odour conditioning exhibited mean learned avoidance of the CS+ odour (figure 2*b*; electronic supplementary material, figure S4). We observed significant correlations in individual learning responses among almost all four conditioning variants in this experiment (0.36 < *r* < 0.59; 1.1 × 10^−7^ < *p* < 2.8 × 10^−3^; figure 2*c*). Two exceptions were MCH+ and 2-heptanone+ (*r* = 0.06; *p =* 0.61) and 2-heptanone+ and 1-pentanol+ in the odour generalization assay replicate (*r* = 0.16; *p =* 0.17), for which we have no confident explanation. These results suggest that individuality in learning performance is largely odour CS- and US-independent and stable over at least 24 h.

A possible explanation of these results is individual variation in US encoding. Flies that receive stronger shocks show stronger learning responses [23], so spontaneous variation in the perception of a US (either shock or bitter taste) may affect the learning responses for many CS. We tested this in the US generalization assay by performing classical and reversal conditioning with OCT and MCH but switching between US within the same animals (figure 2*d*). Both classical and reversal sessions showed significant mean differences in odour preference (figure 2*e*). Comparing across these two aversive US modalities, we observed a positive correlation in learning responses (*r* = 0.59; *p =* 8 × 10^−6^; figure 2*d*). This suggests that in addition to generalizing across CS odorant identity, individual differences in fly learning performance may generalize across aversive US modalities.

## 4. Discussion

Using a training instrument that (i) has versatile control over CS and US and (ii) tracks individual learned behaviour, we observed that flies are idiosyncratic in their learning performance in classical conditioning paradigms. Flies that perform well for one CS/US pair tend to perform well for other CS and US, suggesting that individual differences in learning performance generalize across CS odorants and aversive US modalities. We attempted learning experiments in other modalities (colour as a CS and optogenetic activation of sweet-sensitive neurons as a US) but did not see learning responses, likely a technical failure of our assay. Bees were recently shown to be similarly idiosyncratic, but without generalization between visual and olfactory CS modalities [30]. Our results, in a genetic model organism (see also [31]), provide a basis to probe the mechanistic basis of individuality in learning. Specifically, our results hint that the biological basis for such idiosyncrasy in olfactory learning originates more centrally in the brain than sensory circuit elements dedicated to encoding either CS or US. Stochastic physiological variation [1] in neurons mediating aversive US signals in general could account for individual variation that generalizes across aversive US. Such sites would be ‘loci of individuality' [32] for learning performance. Mushroom body dopaminergic neurons [33–37], particularly those in the protocerebral posterior lateral cluster [9], have been shown to mediate multiple aversive US signals including shock [38,39], bitter taste [40,41] and temperature [42]. Mushroom body output [43–46] and intrinsic neurons [47] are also promising candidates. But valence might also be encoded broadly across multiple populations of neurons [33,48,49], including elements in the sensory periphery [48,49]. Circuit elements known to exhibit high developmental stochasticity [8,50,51] may also be loci of individuality. Our results suggest that flies are a promising model for characterizing the circuit basis of individual variation in generalized learning ability, which is evident even among genetically identical individuals reared in the same environment.

## Data accessibility

All raw data and analysis scripts are available at http://lab.debivort.org/individuality-in-learning and https://zenodo.org/record/4458572. Data were collected by the methods described in the manuscript. Raw values are stored in MATLAB files. Analysis was conducted by MATLAB scripts. Data files and analysis scripts are described individually in html read-me files at http://lab.debivort.org/individuality-in-learning and https://zenodo.org/record/4458572 [29].
